# The Relationships between Sleep and Mental and Physical Health of Chinese Elderly: Exploring the Mediating Roles of Diet and Physical Activity

**DOI:** 10.3390/nu13041316

**Published:** 2021-04-16

**Authors:** Yiqing Zhao, Jianwen Song, Anna Brytek-Matera, Hengyue Zhang, Jinbo He

**Affiliations:** 1 School of Humanities and Social Science, The Chinese University of Hong Kong, Shenzhen 518172, China; yiqingzhao@link.cuhk.edu.cn (Y.Z.); jianwensong@link.cuhk.edu.cn (J.S.); hengyuezhang@link.cuhk.edu.cn (H.Z.); 2 Institute of Psychology, University of Wroclaw, 50-527 Wroclaw, Poland; anna.brytek-matera@uwr.edu.pl

**Keywords:** sleep quality, diet quality, physical activity, physical health, mental health, mediation

## Abstract

Sleep quality, diet quality, and physical activity are significant factors influencing physical and mental health. However, few studies have explored their underlying mechanisms, especially among the elderly population in East Asia, where people have food culture and lifestyles distinct from those living in Western countries. Therefore, the current study aimed to explore the relationships among sleep quality, diet quality, physical activity, and physical and mental health in a Chinese elderly sample. Sleep quality, diet quality, physical activity, physical health, and mental health were investigated among 313 Chinese elderly (aged 51–92 years, M = 67.90, SD = 7.94). Mediation analysis was used to examine the empirical model based on previous theories and literature. Close positive relationships were observed between all factors investigated (*r* = 0.22~0.73, *p* < 0.001). The relationships between sleep quality and physical and mental health were partially mediated by diet quality and physical activity. In clinical interventions, sleep quality, diet quality, and physical activity can be targeted to improve physical and mental health among the older adult populations.

## 1. Introduction

With modern economic and medical developments, human life expectancy has been extended, resulting in rapid growth of aging populations worldwide [1]. China is a country with a particularly large aging population, with 17.4% of its citizens aged 60 and above in 2020 [2]. Due to a culture of caring for the elderly, and increasing attention to health problems, the physical and mental health of the Chinese elderly has become a prominent concern.

Sleep quality, diet quality, and physical activity in the elderly have been receiving extra attention because they have all been shown to be important factors to mental and physical health [3,4,5,6]. Sleep takes up nearly a third of the human lifespan [7] and its quality is especially important to older adults. A number of studies have identified significant changes in sleep structure with aging [8], including age-related changes in sleep patterns (decreased total sleep time and efficiency, lower percentage of rapid eye movement sleep, and less slow-wave sleep) and disturbances (increased night-time spent awake after sleep onset) [9]. In older women, short sleep duration, insomnia, poor sleep quality, and other sleeping disorders have been related to a series of physical and psychological impairments [10]. Sleep deprivation causes oxidative damage in the brain [11,12] and increases lipid peroxidation [13]; it may also contribute to depression, a likely outcome given the known effects of sleep on brain structure, neurogenesis, and hippocampal function [14,15,16]. One recent study showed that sleep quality affected the quality of life among rural older adults in China, with a mediating role of mental health and a moderating role of physical activity [17].

Several studies have found that diet and physical activity are also associated with health status of the elderly [4,18,19,20]. There is a growing body of evidence that certain dietary patterns positively influence health. A healthy, balanced diet consisting of a variety of vegetables and fruits, whole grains, seafood, and nuts, moderate amounts of low-fat dairy products and red meat, and limited quantities of processed foods, saturated and trans fats, have been associated with good mental health across the lifespan [21]. Physical activity has also been found to be related to the health of older adults. There is evidence that regular physical activity in older adults can restore functional capacity (balance, maximal aerobic power, peak muscle force, and flexibility), improve general health, and enhance immune function [22,23]. In addition, moderate-intensity physical exercise is recommended to improve both physical and mental health [24,25].

Although these three factors are clearly related to physical and mental health, the underlying mechanisms through which they interact remain unclear. Since previous investigations have revealed the close relationships between sleep quality and diet quality as well as between diet quality and overall health, we propose a mediation model in which sleep quality affects physical and mental health through the mediating roles of diet quality and physical activity.

### 1.1. The Mediating Role of Diet Quality in the Relationships between Sleep and Health in Older Adults

Diet is a vital determinant of physical health. A poor diet containing high levels of fat or sugar has been shown to increase the risk for obesity [26]. On the other hand, diets such as DASH (Dietary Approaches to Stop Hypertension) or the Mediterranean diet have been associated with relief of hypertension [27] and reduced inflammatory markers [28], respectively. Moreover, prior studies have shown that depressive symptoms are negatively associated with the consumption of fish, vegetables, olive oil, and cereal, and positively related to the consumption of sweets [29].

Based on previous studies [30,31,32,33], we hypothesize that diet is an important mediator of the relationship between sleep and health, which includes both the physical and mental aspects. Diet quality was considered as a potential confounding variable by most of the previous studies, but a recent systematic review provided evidence that diet mediates the relationships between sleep and obesity, cardiovascular health, metabolic disease, and major depression [30,31]. For example, short and poor-quality sleep was related to cardiometabolic disease and mediated by imbalanced diet, which led to dysregulation of appetite-related hormones and elevated activity in reward-related brain regions [32,33]. Insufficient sleep was also found to be related to excess energy intake via increased snacking and the number of meals consumed per day due to hedonic factors [34]. A cross-sectional observational study of female adolescents found that sufficient and uninterrupted sleep was related to a well-balanced diet and a more favorable hormonal profile [35]. Thus, we hypothesize a mediating role of diet quality on the relationship between sleep quality and physical and mental health among older Chinese adults.

### 1.2. The Mediating Role of Physical Activity in the Relationships between Sleep and Health in Older Adults

Physical activity plays a critical role in both physical and mental health. Regular physical activity can help to prevent excess weight gain or regain, as well as to achieve and maintain a healthy weight [36]. Interventions targeting physical activity can be used to manage and prevent obesity [26]. Regular physical activity has proven useful in the primary and secondary prevention of numerous chronic diseases (including cardiovascular disease, obesity, cancer, and hypertension) with risk reductions as high as 20–30% [37]. Moreover, regular physical activity has been associated with longer overall life expectancy as well as with disability-free and quality-adjusted life expectancy, which indirectly suggests that physical activity endows health benefits [23]. Increased amounts of physical activity that is moderate-to-vigorous in intensity, and decreased amounts of sedentary behavior, have been associated with lower levels of depression in overweight and obese adults [31,38].

Given these findings, we hypothesize that physical activity offers another potential mechanism underlying the relationship between sleep quality and mental and physical health. Daily physical activity of at least 60 min has been positively associated with sleep quantity [39]. Another study showed that sleep quality was related positively to physical activity and negatively to sedentary behavior [26]; this makes sense given that high-quality, uninterrupted sleep can make individuals feel relaxed and energetic, encouraging them to participate in physical activity rather than sedentary behaviors [26]. Taking into account the impacts of physical activity on mental health, we hypothesize a mediating role of physical activity on the relationship between sleep quality and physical and mental health among older Chinese adults.

### 1.3. The Current Study

Previous studies focusing on sleep, diet, and physical activity have three major limitations. First, although numerous studies have confirmed that sleep, diet, and physical activity are related to health, we found no previous research into the possible mechanisms among these variables. Therefore, we propose a mediation model to identify and explain such a mechanism. Second, the majority of existing studies were conducted among children and youth [40,41,42,43]. Due to the increasing number of aging populations worldwide [1], it is critical to also investigate the relationships among sleep, diet, physical activity, and health in elderly people. Previous research has shown that sleep disorders have a high incidence (47.2%) in older Chinese adults and seriously impact their quality of life [44]. Most bodily functions deteriorate progressively with age. Sleep disturbances and complaints (e.g., insomnia, drowsiness) are common in older adults and predict poor physical and mental health [45]. Therefore, age-related changes in sleep should be investigated as targets for health-promoting interventions in this population. Third, the majority of prior studies were carried out in Western countries (e.g., United Kingdom, United States of America) and few in East Asia [43,46,47,48]. Dietary and other behaviors cannot be separated from the sociocultural background [49]. Therefore, due to differences in geographical location, culture, and lifestyle, the existing findings may not be applicable to the older Chinese population. To the best of our knowledge, there is only one study showing that mental health partially mediates the effect of sleep quality on quality of life among rural elderly in China, and that physical activity moderates the effect of mental health on the relationship between sleep quality and quality of life [17]. No studies have directly examined the association between sleep quality and health in older Chinese adults.

**Figure 1:**
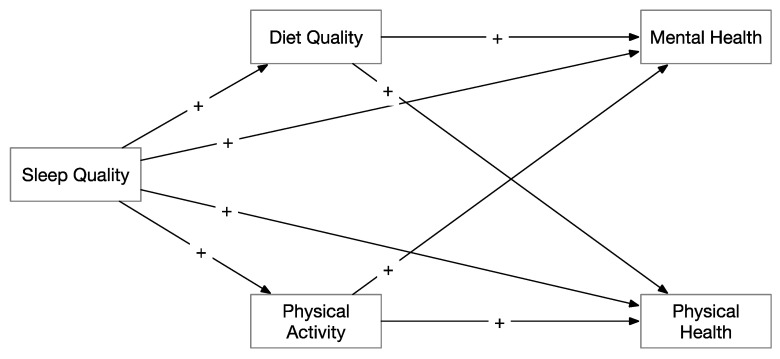
Conceptual model of the mediation analysis for both mental health and physical health. Note: + positive prediction.

Previous research has provided evidence for a relationship between sleep quality and physical and mental health, but the underlying mechanisms remain unclear. Therefore, the objective of the present study was to identify the connection between sleep quality and health (both physical and mental) by examining two potential mediators: diet quality and physical activity. We hypothesized that associations between all variables would be positive among older Chinese adults, and the relationships between sleep quality and physical and mental health would be mediated by overall diet quality and physical activity (see Figure 1). Because most of the relevant research has been conducted in young people in Western countries, in this study an elderly Chinese sample was used with the aim of overcoming these limitations. We hypothesized that better sleep quality was positively associated with overall diet quality and physical activity, which was in turn positively linked to both physical and mental health in older Chinese adults.

## 2. Materials and Methods

### 2.1. Participants

By employing both the convenience sampling technique (e.g., research assistants went to elderly activity centers) and the snowball sampling technique (e.g., existing participants provide referrals to participate in the project), we initially investigated 320 elderly individuals from three Chinese cities: Shenzhen and Guangzhou (South) and Qiqihar (North). After removing incomplete surveys, 313 (48.2% male) subjects were retained, aged 51 to 92 years old (M = 67.90, SD = 7.94). Body mass index (BMI) of our sample ranged from 13.67 to 36.75 kg/m^2^ (M = 22.70, SD = 3.36). Using BMI standards for Chinese adults [50], 10.5% of the subjects were classified as underweight (BMI < 18.5 kg/m^2^), 57.8% were normal weight (18.5–23.9 kg/m^2^), 26.2% were overweight (24–27.9 kg/m^2^), and 5.4% were obese (BMI ≥ 28 kg/m^2^). Additional demographic information, including education level, marital status, urban/rural residence, ethnicity, hypertension status, and diabetes status, is presented in Table 1.

### 2.2. Data Collection

The present study was approved by the Institutional Review Board of the Chinese University of Hong Kong, Shenzhen (No. 1-PSY-H). To be consistent with previous studies on elderly Chinese [51], inclusion criteria required all participants to be over 50 years old. Interviews were conducted by research assistants (YZ & HZ) who were trained by their supervisor and by healthcare professionals on study procedures, data collection, and ethics. Along with the paper-and-pencil questionnaires, participants received an informed consent form. After giving their consent to be part of the study, participants were asked if assistance was needed (e.g., for questionnaires to be read aloud in cases of visual impairment). In our sample, 126 (40.3%; *M*_age_ = 67.94 ± 8.77) participants completed the interviews with help from the research assistants and 187 (59.7%; *M*_age_ = 67.88 ± 7.36) participants completed the survey independently. There was no significant difference between these groups with regard to age (*t* = 0.06, *p* = 0.948). Upon completion of the questionnaires, each participant received a gift worth about 10 ¥ ($1.41). The current study is a part of a project about eating and body image among Chinese elderly, a paper has been published based on the same sample [52].

### 2.3. Measures

As well-documented in the empirical literature [53], the length of the survey can greatly increase response burden, decrease the response rates, and damage the validity of the survey. Considering the decreased cognitive function, attention span, and vigor of older adults [54,55], we decided to use the equivalent single-item or short alternatives instead of the full-length scales to measure sleep quality, diet quality, physical activity, and mental and physical health.

**Sleep quality.** The Chinese version of the single-item Sleep Quality Scale (SQS) was used [56]. The SQS enables a patient-reported rating of sleep quality over a 7-day recall period using a visual analog scale [57]. Based on the integer score from 0 to 10, sleep quality can be divided into 5 categories: 0 = “terrible”, 1 to 3 = “poor”, 4 to 6 = “fair”, 7 to 9 = “good”, and 10 = “excellent”. To assess sleep quality, respondents were instructed to consider the following core components: *how many hours of sleep they had, how easily they fell asleep, how often they woke up during the night (except to go to the bathroom), how often they woke up earlier than they had to in the morning, and how refreshing their sleep was*. The SQS demonstrated good validity by showing strong correlations (*r* = −0.76 to −0.92) with other established sleep quality scales such as the Pittsburgh Sleep Quality Index [58]. The SQS also presented acceptable to good test-retest reliability with Intraclass correlation coefficients from 0.55 to 0.74 as shown in the previous studies [57].

**Diet quality.** The Self-Rated Diet Quality Measure (SRDQM) is a single-item self-reported measure of overall diet quality, using a 5-point response scale (from 1 = “excellent” to 5 = “poor”) [59]. The SRDQM demonstrated excellent construct validity by showing significant associations with both subjective and objective dietary intake (e.g., fruit and vegetable intake), eating behaviors (e.g., frequency of fast-food dining), and related health outcomes (e.g., blood pressure) [59]. By using the SRDQM, a study also showed acceptable test-retest reliability (Cohen’s κ = 0.55) of the SRDQM, and the authors further concluded that self-reported diet quality was a useful indicator of an overall diet quality of a population [60]. In the present study, the Chinese version of the SRDQM was used, and it was obtained based on standard translation and back-translation procedures [61].

**Physical activity.** A single-item physical activity screening tool [62] was used. Participants were asked to report the number of days in the past week in which they exercised for at least 30 min. In this context, exercise included sport, jogging, biking, and similar activities, and excluded housework and activities that may be part of the participant’s job [62]. An open-response scale was used, with valid responses ranging from 0 to 7 days. This new single-item physical activity measure has demonstrated good test-retest reliability (Cohen’s κ = 0.76) and good validity by showing a positive correlation (*r* = 0.53) with the Global Physical Activity Questionnaire [62]. In the present study, the Chinese version was used and it was obtained based on standard translation and back-translation procedures [61].

**Mental and physical health.** We used four items from the PROMIS Global Health brief measure which was developed by the National Institutes of Health to assess global physical health and mental health [63]. Specifically, two items were used to assess physical health: “*In general, how would you rate your physical health?*” and “*To what extent are you able to carry out your physical activities such as walking, climbing stairs, carrying groceries, or moving a chair?*”. Another two items were used to assess mental health: “*In general, how would you rate your mental health, including your mood and your ability to think?*” and “*In general, how would you rate your satisfaction with social activities and relationships?*”. All items were answered using a 5-point Likert scale ranging from 1 “excellent”/“completely” to 5 “poor”/“not at all”. The previous study showed that Cronbach’s α was 0.73 and 0.81 for the physical health and mental health scales, respectively [63], indicating good internal consistency of the measure. Moreover, it also had good construct validity by showing significant correlations with health-related variables (e.g., physical function, fatigue, anxiety, and depressive symptoms) [63]. In the present study, the Chinese version was used, and it was obtained based on standard translation and back-translation procedures [61] (Cronbach’s α_physical health_ = 0.61; Cronbach’s α_mental health_ = 0.89 in the current sample).

### 2.4. Statistical Analysis

All statistical analyses were performed using R software version 4.0.0. Mediation analysis was conducted using the R package “lavaan” [64]. All variables were standardized prior to analysis. We used a dummy variable for sex where 0 = “*male*” and 1 = “*female*”. We examined relationships between variables using Pearson’s bivariate correlations. For correlation coefficients, a value of 0.10, 0.30, and 0.50 is considered small, medium, and large, respectively [65] Significance of the indirect effect was based on its 95% confidence interval (CI) through bootstrapping (based on 10,000 bootstrap samples). Sex, age, and BMI were controlled in the analysis.

## 3. Results

### 3.1. Descriptive and Correlation Analysis

Descriptive statistics are shown in Table 2. Correlation coefficients between each pair of variables are shown in Table 3. Specifically, all study variables were positively related to each other, with correlation coefficients ranging from *r* = 0.22 (*p* < 0.001; between sleep quality and diet quality) to *r* = 0.73 (*p* < 0.001; between mental health and physical health).

### 3.2. Mediation Analysis

**Figure 2:**
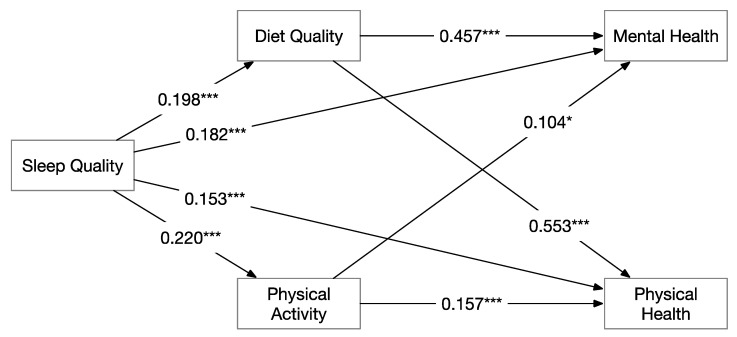
Regression models of the mediation analysis for both mental health (*R*^2^ = 0.356) and physical health (*R*^2^ = 0.453). Note: * *p* < 0.05, *** *p* < 0.001.

The mediating role of diet quality and physical activity on the relationship between sleep quality and mental and physical health were tested, controlling for sex, age, and BMI (See Figure 2).

### 3.3. Mental Health

As shown in Table 4, diet significantly mediates the relationship between sleep quality and mental health (total indirect effect = 0.091; 95% CI = 0.038–0.154). In contrast, the effect of physical activity was not statistically significant (total indirect effect = 0.023; 95% CI = 0.004–0.053). The mediation model explained 35.6% of the variance in mental health.

### 3.4. Physical Health

As shown in Table 5, both diet and physical activity significantly mediated the relationship between sleep quality and physical health. The indirect effect for diet was 0.110 (95% CI = 0.047–0.177) and for physical activity was 0.035 (95% CI = 0.014–0.068). The mediation model explained 45.3% of the variance in physical health.

## 4. Discussion

The aim of the present study was to identify whether and to what extent the relationships between sleep quality and physical and mental health are mediated by diet quality and physical activity among elderly Chinese. Results confirmed our hypotheses by revealing that the relationships between sleep quality and physical and mental health were partially mediated by diet quality and physical activity.

In the present study we observed positive correlations between all factors: sleep quality, diet quality, physical activity, physical health, and mental health. One possible explanation for this is that high-quality sleep gives older adults the energy to engage in regular physical activity and thus improve their physical health. Regular physical activity is beneficial in preventing chronic diseases and their recurrence, enhancing immunity, and improving whole-body physical function [23]. Physical activity may contribute to older adults’ mental health by reducing anxiety and depression, transiently increasing cerebral blood flow, increasing the activity of reticular formation neurons, increasing the secretion of catecholamines, and facilitating the passage of arousing chemicals across the blood–brain barrier [23]. In contrast, disordered sleep and sedentary behaviors have been shown to increase the risk of many health problems [66]. Previous studies have reported that physical inactivity and depressed mood are associated with insomnia, sleep-disordered breathing, and excessive daytime sleepiness in older adults [67,68]. Therefore, the elderly and their carers should prioritize high-quality sleep, a healthy, balanced diet, and regular physical activity to improve overall wellbeing.

Mediation analysis indicated that diet quality plays a significant mediating role between sleep quality and physical and mental health. This finding is consistent with the results of previous studies in children and adults and extends the evidence to an elder sample [32,34,36,69,70,71]. It is worth noting that the correlation coefficients observed in the current study were much higher than those from in children and young adults (e.g., in our work, the correlation coefficient between diet and mental health and physical health is 0.637 and 0.545, respectively), suggesting that diet quality might be a stronger mediator among the elderly. This may be explained in part by the fact that metabolism declines with age, so older adults would require a well-balanced diet as well as sufficient sleep in order to improve their health.

Interestingly, we found physical activity only plays a significant mediating role between sleep quality and physical health but plays a marginal mediating role between sleep and mental health. This indicates that compared to the effect of physical activity on physical health and the effects of diet quality on both mental and physical health, the effect of physical activity on mental health is much smaller. With the aging process [72], older adults are having more difficulty to do physical activities that are intense enough to improve mental health; however, keeping a balanced diet is less likely to be limited by their decreasing in physical functioning due to aging.

Overall, the current study revealed that sleep quality may affect physical and mental health via diet quality and physical activity among Chinese elderly, and confirmed the hypothesis that sleep quality is associated with well-being in older adults from an Eastern culture [17]. Considering that the proposed model explained large amounts of variance for both mental and physical health (35.6% and 45.3%, respectively), the present findings may help to elucidate factors contributing to older adults’ health, thereby facilitating the development of targeted interventions. It is worth pointing out that older Chinese adults comprise a large population that is known to have sleep and health problems [44]. Since previous studies have shown that approximately 47.2% of elderly people in China experience sleep disorders and poor sleep quality, which negatively affects quality of life and subjective well-being [44], interventions aimed at improving sleep quality of the elderly could also lead to positive changes to diet quality, physical activity, and physical and mental health.

As with the majority of studies, the current study is subject to limitations. First, given the cross-sectional design, the results cannot be interpreted in a causal way. Second, the measures used were self-report scales. If we used the objective methods, the results might be different; thus, to confirm our findings, future researchers should consider using objective measures to assess the variables (e.g., using accelerometer to measure physical activity [73], smartwatches to measure sleep quality [74], and real food intake to measure diet quality [75]). Third, the use of convenience sampling and snowball sampling may have introduced some sampling bias. Finally, the use of short measures (e.g., single-item measures for assessing sleep, diet quality and physical activity) may also introduced bias to the results, as the constructs measured by single-item measures and the original full measures might be different. However, it should be noted that whether the original full measures are better than single-item measures is still under debate [76,77,78]. Despite these limitations, the present study found that diet quality and physical activity partially mediated the association between sleep quality and physical and mental health among Chinese elderly.

## 5. Conclusions

The current study showed that sleep quality had positive relationships with both physical and mental health among Chinese elderly, and the relationships were partially mediated by diet quality and physical activity. This study provided useful data on the elderly in China, which is an ever-growing population and one that is understudied from a psychological perspective. Comprehensive psychological and behavioral interventions for improving sleep quality, diet quality, and physical activity will be beneficial to older adults’ well-being. Future studies should examine the long-term effects of these interventions among elderly people in China.

## Figures and Tables

**Table 1:** Demographic information of the participants.

	Males (*N* = 151)	Females (*N* = 162)	Overall Percent (%)
Education			
Primary school or less	15	23	12.4
Junior high	29	44	23.8
Senior high	81	67	48.2
Undergraduate	22	22	14.3
Postgraduate or above	4		1.3
Marriage			
Married	132	113	78.5
Other	19	48	21.5
Residence			
City	120	114	75.0
Rural	31	47	25.0
Ethnicity			
Han	129	111	76.7
Minorities	22	51	23.3
Hypertension			
Yes	71	65	43.6
No	79	97	56.4
Diabetes			
Yes	39	45	26.9
No	111	117	73.1

**Table 2:** Means (*M*), standard deviations (*SD*) of the study variables.

	*M/n%*	*SD*
Sex (1 = male; 2 = female)	48.2 (males)	-
Age	67.9	7.95
BMI	22.70	3.36
Sleep quality Diet quality Physical activity Mental health Physical health	7.06	2.58
	3.23	1.02
	5.08	2.39
	48.29	9.77
	45.72	9.21

**Table 3:** Correlations between variables.

Variable	1	2	3	4	5	6	7	8
1. Sex	1							
2. Age	−0.058	1						
3. BMI	−0.126 *	0.005	1					
4. Sleep quality	−0.073	0.068	−0.086	1				
5. Diet quality	0.010	−0.032	−0.074	0.216 ***	1			
6. Physical activity	−0.014	−0.004	−0.217 ***	0.226 ***	0.240 ***	1		
7. Mental health	−0.072	0.032	−0.202 ***	0.319 ***	0.637 ***	0.332 ***	1	
8. Physical health	−0.058	0.068	−0.224 ***	0.317 ***	0.545 ***	0.265 ***	0.729 ***	1

Note. * *p* < 0.05, *** *p* < 0.001.

**Table 4:** Pathways of direct and indirect effects for mental health.

	Point Estimates	SE	Bootstrapping 95%CI	
			Lower	Upper
Direct effect	0.182 ***	0.053	0.079	0.291
Path 1				
Sleep quality	0.091 **	0.03	0.038	0.154
↓				
Diet quality				
↓				
Mental health				
*Path 2* #				
Sleep quality	0.023	0.012	0.004	0.053
↓				
Physical activity				
↓				
Mental health				
Total effect	0.296 ***	0.056	0.184	0.405

Note: ** *p* < 0.01, *** *p* < 0.001; SE = standardized error; CI = confidence interval. # In path 2, the indirect effects are inconsistent as shown in point estimate and in the bootstrapping 95%CI. Specifically, in the point estimate, the *p* value for the indirect effect of 0.023 is 0.058 which is less than 0.05 (i.e., nonsignificant); however, the bootstrapping 95%CI does not contain 0, indicating a significant indirect effect. Considering the relatively small indirect effect and the marginal *p* value, we did not consider the path 2 as a significant indirect path even though the three valuables (sleep quality, physical activity, and mental health) presented significant associations in the bivariate correlation analysis (Table 3).

**Table 5:** Pathways of direct and indirect effects for physical health.

	Point Estimates	SE	Bootstrapping 95%CI	
			Lower	Upper
Direct effect	0.153 ***	0.048	0.056	0.246
*Path 1*				
Sleep quality	0.110 ***	0.033	0.047	0.177
↓				
Diet quality				
↓				
Mental health				
*Path 2*				
Sleep quality	0.035 **	0.013	0.014	0.068
↓				
Physical activity				
↓				
Mental health				
Total effect	0.297 ***	0.056	0.190	0.408

Note: ** *p* < 0.01, *** *p* < 0.001; SE = standardized error; CI = confidence interval.

## Data Availability

The dataset used during the current study are available from the corresponding author on reasonable request.
